# Differential impact of anemia in relation to sex in patients with myocardial infarction

**DOI:** 10.3389/fcvm.2023.1108710

**Published:** 2023-02-23

**Authors:** Vojko Kanic, Gregor Kompara, David Suran

**Affiliations:** Division of Internal Medicine, Department of Cardiology and Angiology, University Medical Center Maribor, Maribor, Slovenia

**Keywords:** anemia, myocardial infarction, sex, PCI, outcome, mortality

## Abstract

**Background:**

Data on the possible sex-specific effects of anemia on clinical outcome in patients with myocardial infarction are extremely sparse, conflicting, and inconclusive. We investigated the possible sex-specific effects of anemia on outcome in patients with myocardial infarction (MI) who underwent percutaneous coronary intervention (PCI).

**Methods:**

Data from 8,318 patients, who were divided into four groups: men and women with and without anemia on admission, were analyzed. The association between anemia and sex and 30-day and long-term mortality was assessed. The median follow-up time was 7 years (25th, 75th percentile: 4, 11).

**Results:**

Non-anemic men had the lowest 30-day and long-term observed mortality (4.3, 18.7%), followed by non-anemic women (7.0, 25.3%; p < 0.0001, p < 0.0001). Anemic men and women had similar mortality rates (12.8, 46.2%) and (13.4, 45.6%; p = 0.70, p = 0.80), respectively. The anemia/sex groups were independently associated with 30-day and long-term mortality (p = 0.033 and p < 0.0001, respectively). Compared to non-anemic men, non-anemic and anemic women had a similar risk of death at 30 days, but anemic men had a 50% higher risk of death (OR 1.12; 95% CI 0.83–1.52; p = 0.45, OR 1.30; 95% CI 0.94–1.79; p = 0.11, OR 1.50; 95% CI 1.13–1.98; p = 0.004, respectively). In the long term, anemic men had a 46% higher, non-anemic women 15% lower, and anemic women a similar long-term mortality risk to non-anemic men (HR 1.46; 95% CI 1.31–1.63; p < 0.0001, HR 0.85; 95% CI 0.76–0.96; p = 0.011, and HR 1.06; 95% CI 0.93–1.21; p = 0.37, respectively).

**Conclusion:**

Our result suggests that the influence of anemia in patients with MI is different in men and women, with anemia seemingly much more harmful in male than in female patients with MI.

## Introduction

The prevalence of anemia is estimated to be nearly one-quarter of the population worldwide, with women (especially younger women) more commonly affected than their male peers (1–4). The prevalence of anemia in patients with myocardial infarction (MI) varies from 6.4 to 43%, depending on the definition of anemia and patient characteristics (5–7). As in the normal population, the prevalence of anemia in patients with MI is higher in younger women than in their male peers, but this difference usually disappears after the age of 60 years (8).

Most evidence also supports an association between anemia and the development of coronary artery disease or poor outcome in patients with coronary artery disease, yet there are limited data describing the role of sex in the association between anemia and outcomes (7, 9–11). Differential effects of anemia in men and women in relation to the occurrence of coronary artery disease have been found (9). Some investigators have found an association between anemia and coronary artery disease in women but not in men (11, 12), whereas others have found the opposite (13). There is substantial evidence that anemia is associated with adverse outcomes in patients with MI (6, 14–18). Data on the possible sex-specific effects of anemia on clinical outcome in patients with myocardial infarction are extremely sparse, conflicting, and inconclusive (6–8, 19). Anemia has been found to be associated with mortality only in men (7), or detrimental to both sexes (6, 8, 17, 19). We hypothesized that because of sex-specific biology (20–22), different risk factor profiles (23), unusual pathophysiological mechanisms of coronary artery disease in women (22), and gender-specific (culturally determined) psychosocial stressors (22), the effects of anemia in patients with myocardial infarction might be different in men and women MI. The aim of our study was to investigate the possible sex-specific effects of anemia on outcome in patients with MI who underwent percutaneous coronary intervention (PCI).

## Materials and methods

A retrospective single-center analysis of prospectively collected data was conducted at the University Medical Centre Maribor, a tertiary institution with a 24/7 PCI service. We screened all 8,343 consecutive patients with MI treated with PCI between January 2007 and December 2018. Patients with missing hemoglobin data (25, 0.4%) were excluded. The final study population comprised 8,318 patients whose baseline characteristics, clinical risk factors, and treatment characteristics were obtained from hospital electronic medical records. Group hemoglobin data were provided for all patients, and data on all other essential patient and procedural characteristics were at least 99% complete. Ascertainment of end points was 100% complete. Long-term mortality was assessed over a median period of 7 years (25th, 75th percentile: 4, 11) up to 1 August 2021. Data on the dates of death were provided by the Slovenian National Cause of Death Registry. The study was approved by the Local Institutional Ethics Committee (UKC-MB-KME-59/19), and all methods were performed following relevant guidelines and regulations.

### Definitions

Anemia was defined according to World Health Organization recommendations: a serum hemoglobin level of <130 g/L for men and <120 g/L for women (24). The hemoglobin level was determined at admission. The MI was confirmed in patients by a history of chest pain, diagnostic electrocardiographic changes, and serial elevations of cardiac biomarkers (above the 99th percentile URL in our laboratory) according to published guidelines (25–27). The Bleeding Academic Research Consortium (BARC) 3a bleeding criteria were used (28). Renal dysfunction was defined as a glomerular filtration rate (GFR) of less than 60 ml/kg/1.73 m^2^. The ventricular ejection fraction was assessed by bedside echocardiography in the first 48 h after admission. Heart failure was defined according to clinical criteria (bilateral pulmonary rales, S_3_ gallop, edema) and/or pulmonary edema on chest X-ray and/or ejection fraction <30%. The diagnosis of cardiogenic shock (CS) was made based on the accepted definition of a systolic blood pressure ≤90 mm Hg for ≥30 min or the need for supportive measures to maintain a systolic blood pressure of >90 mm Hg, clinical signs of pulmonary congestion, and signs of end-organ hypoperfusion.

### End points

The primary end point was 30-day, and the secondary end point was long-term all-cause mortality in anemic and non-anemic male and female patients.

### Statistical methods

The patients were divided into four groups: men and women with and without anemia on admission. These groups were compared. The Kolmogorov–Smirnov test was used to assess normal distribution. Differences between groups in baseline clinical, angiographic, and procedural characteristics were compared with the Jonckheere-Terpstra test for continuous variables and the Chi-square test or Fischer’s exact test for categorical variables. We counted end point events that occurred during the follow-up period and compared their rates between non-anemic/anemic men and women. The cumulative incidence rates of the unadjusted long-term mortality were estimated by the Kaplan–Meier method and compared by the log-rank test. Binary logistic regression models were performed using the Enter mode to determine the possible association between anemia and sex and 30-day mortality, and Cox regression analysis was used to determine hazard ratios (HR) as estimates of long-term mortality. The models were adjusted for age, hypertension, diabetes, hyperlipidemia, renal dysfunction on admission, ST-elevation MI, cardiogenic shock on admission, TIMI 0/1 after PCI, bleeding, P2Y12 receptor antagonists, and sex-specific non-anemic/anemic groups (men and women with and without anemia on admission). HRs were calculated using a model stratified by these groups. The variables included and retained in the model were based on previous literature reports and experience that these factors are known to influence all-cause mortality. All included variables had a univariable association with 30-day mortality p < 0.002 and a variance inflation factor (VIF) <1.4. We calculated adjusted HRs for all four groups. The group of non-anemic men (Hb ≥130 g/L) was used as the reference group. Only cardiogenic shock on admission was included as a covariate in the calculations. In addition, we ran regression models for each sex separately, including anemic/non-anemic men or women as a variable in the model in addition to the other variables listed above. Data were analyzed with SPSS 25.0 software for Windows (IBM Corp., Armonk, NY, USA). All p-values were two-sided, and values less than 0.05 were considered statistically significant.

## Results

Anemia was found in 2,319 (27.9%) patients at admission, 1,422 (17.4%) anemic males, 897 (10.8%) anemic females, 4,290 (51.6%) non-anemic males, and 1,709 (20.5%) non-anemic females. Patients with anemia were more often female, older, had diabetes, worse renal function, lower cholesterol, and lower ejection fraction. Anemic patients were more likely to have more extensive and severe coronary artery disease (more PCI of the left main coronary artery). They were also more likely to present with cardiogenic shock and more likely to suffer heart failure after PCI. They were more likely to have had femoral access performed, and although they received P2Y12 receptor antagonists less frequently, they were more prone to bleeding and PCIs were less successful. Both 30-day and long-term mortality were higher in anemic patients. Baseline data and procedural characteristics of the study population are shown in Table 1. Baseline characteristics of the four groups based on sex and anemia are shown in Table 2. The groups differed with respect to age, GFR, presence of diabetes, hypertension, hyperlipidemia, ST-elevation MI and cardiogenic shock on admission, PCI of different coronary arteries, the success rate of PCI, ejection fraction, heart failure after PCI, the bleeding rate, and the use of P2Y12.

**TABLE 1:** Baseline and procedural characteristics of the study population.

Variable	Non-anemic PTS	Anemic PTS	Population	p
	n = 5,999	n = 2,319	n = 8,318	
Age (years)	63.0 (11.8)	70.2 (11.3)	65.0 (12.2%)	<0.0001
Women	1,709 (23.5%)	897 (38.7%)	2,606 (31.3%)	<0.0001
Diabetes mellitus	1,303 (21.7%)	716 (30.9%)	2,019 (24.3%)	<0.0001
Hypertension	3,441 (57.4%)	1,327 (57.2%)	4,768 (57.3%)	0.92
Hyperlipidemia	2,875 (47.9%)	763 (32.9%)	3,638 (43.7%)	<0.0001
STEMI	3,337 (55.6%)	1,403 (60.5%)	4,740 (57.0%)	0.50
Cardiogenic shock	241 (4.0%)	170 (7.3%)	411 (4.9%)	<0.0001
GFR (ml/min/1.73 m^2^)	84.4 (67.9, 100.8)	66.8 (45.2, 89.4)	80.8 (61.3, 98.8)	<0.0001
EF (%)	47.4 (7.3)	45.2 (8.9)	46.8 (7.9)	<0.0001
Heart failure	569 (9.5%)	395 (17.0%)	963 (11.6%)	<0.0001
Renal dysfunction	961 (16.1%)	996 (43.0%)	1,959 (23.6%)	<0.0001
PCI radial access	2,136 (35.6%)	519 (22.4%)	2,651 (31.9%)	<0.0001
PCI LMCA	162 (2.7%)	112 (4.8%)	274 (3.3%)	<0.0001
PCI LAD	2,401 (40.0%)	802 (34.6%)	3,203 (38.5%)	<0.0001
PCI LCX	1,377 (23.0%)	473 (20.4%)	1,850 (22.2%)	0.011
PCI RIGHT	1,760 (23.9%)	756 (32.6%)	2,516 (30.2)	0.004
TIMI 0/1 after PCI	346 (5.8%)	204 (8.8%)	550 (6.6%)	<0.0001
P2Y12	5,383 (89.7%)	1,996 (86.1%)	7,379 (88.7%)	<0.0001
Bleeding	338 (5.6%)	320 (13.2%)	658 (7.9%)	<0.0001
**Mortality outcome**				
Mortality 30-day	305 (5.1%)	302 (13.0%)	607 (7.3%)	<0.0001
Long-term death	1,235 (20.6%)	1,066 (46.0%)	2,301 (27.7%)	<0.0001

Data are expressed as mean ± SD, a number (percentage), or the median (interquartile range). BMI, body mass index; %BF, body fat percentage; DAPT, dual antiplatelet therapy; EF, ejection fraction; GFR, glomerular filtration rate; HDL, HDL-cholesterol; LAD, left anterior descending artery; LCX, circumflex artery; LDL, LDL-cholesterol; LMCA, left main coronary artery; PCI, percutaneous intervention; Right, right coronary artery; STEMI, ST-elevation myocardial infarction; PTS, patients; TIMI, thrombolysis in myocardial infarction.

**TABLE 2:** Baseline and procedural characteristics of the study population classified by anemia and sex.

	Four anemia-sex groups						
Variable	Anemic men		Anemic women	Non-anemic men		Non-anemic women	p
	n = 1,422	p	n = 897	n = 4,290	p	n = 1,709	
Age (Years)	68.9 (10.8)	<0.0001	72.4 (11.7)	60.9 (11.4)	<0.0001	68.2 (11.3)	<0.0001
Hemoglobin (g/L)	120 (110, 125)	<0.0001	110 (101, 115)	143 (134, 151)	<0.0001	128 (120,135)	<0.0001
GFR (ml/min/1.73 m^2^)	69.2 (47.2, 95.0)	<0.0001	59.8 (41.1, 83.9)	86.9 (71.4, 103.4)	<0.0001	76.9 (59.4, 93.1)	<0.0001
EF (%)	45.2 (8.9)	0.96	45.1 (9.1)	47.7 (7.0)	<0.0001	46.8 (8.0)	<0.0001
**Categorical variables**							
		**Anemic men (%)**		**Anemic women (%)**		**Non-anemic men (%)**	**Non-anemic women (%)**
PCI radial access		319 (22.4)		200 (22.3)		1,546 (36.0)	590 (34.5)
Diabetes mellitus		413 (29.0)		303 (33.8)		886 (20.7)	417 (24.4)
Hypertension		777 (54.5)		550 (61.3)		2,328 (54.3)	1,113 (65.1)
Hyperlipidemia		453 (31.9)		310 (64.6)		2,140 (49.9)	735 (43.0)
Cardiogenic shock		196 (7.5)		64 (7.1)		156 (3.6)	85 (5.0)
TIMI 0/1 after PCI		132 (9.3)		72 (8.0)		249 (5.8)	97 (5.7)
STEMI		881 (62.0)		522 (58.2)		2,410 (56.2)	927 (54.2)
LMCA		62 (4.4)		50 (5.6)		110 (2.6)	52 (3.0)
LAD		484 (34.0)		318 (35.5)		1,676 (39.1)	725 (42.4)
LCX		290 (20.4)		183 (20.4)		1,018 (23.7)	359 (21.0)
Right		446 (31.4)		310 (34.6)		1,303 (30.4)	456 (26.7)
P2Y12		1,226 (86.2)		770 (85.8)		3,882 (90.5)	1,501 (88.7)
Bleeding		170 (12.0)		150 (16.7)		217 (5.1)	121 (7.1)
Heart failure		238 (16.7)		157 (17.5)		360 (8.4)	208 (12.2)
Death, 30-day		182 (12.8)		120 (13.4)		185 (4.3)	120 (7.0)
Death, long-term		657 (46.2)		409 (45.6)		803 (18.7)	432 (25.3)
**Comparison of categorical variables**							
		**Anemic men vs. anemic women**	**Anemic men vs. non-anemic men**	**Anemic men vs. non-anemic women**	**Anemic women vs. non-anemic men**	**Anemic women vs. non-anemic women**	**Non-anemic men vs. non-anemic women**
Radial access		NS	<0.0001	<0.0001	<0.001	<0.0001	NS
Diabetes		0.016	<0.0001	0.003	<0.0001	<0.0001	0.002
Hypertension		0.002	NS	<0.0001	<0.0001	NS	<0.0001
Hyperlipidemia		NS	<0.0001	<0.0001	<0.0001	<0.0001	<0.0001
TIMI 0/1 after PCI		NS	<0.0001	<0.0001	0.015	0.024	NS
STEMI		NS	<0.0001	<0.0001	NS	NS	NS
Cardiogenic shock		NS	<0.0001	0.004	<0.0001	0.026	0.02
LMCA		NS	<0.0001	NS	<0.0001	0.002	NS
LAD		NS	0.001	<0.0001	0.045	0.001	0.018
LCX		NS	0.01	NS	0.033	NS	0.025
Right		NS	NS	<0.0001	0.016	<0.0001	0.004
P2Y12		NS	<0.0001	NS	<0.0001	NS	0.003
Bleeding		0.001	<0.0001	<0.0001	<0.0001	<0.0001	0.003
Heart failure		NS	<0.0001	<0.0001	<0.0001	<0.0001	<0.0001
Death, 30-day		NS	<0.0001	<0.0001	<0.0001	<0.0001	<0.0001
Death, long-term		NS	<0.0001	<0.0001	<0.0001	<0.0001	<0.0001

Values are mean ± SD or %. Categorical variables were compared using the Chi-square test. GFR, glomerular filtration rate; EF, ejection fraction; LAD, left anterior descending artery; LCX, circumflex artery; LMCA, left main coronary artery; P2Y12, P2Y12 receptor antagonists; PCI, coronary intervention; Right, right coronary artery; STEMI, ST-elevation myocardial infarction; TIMI, thrombolysis in myocardial infarction.

### Mortality

#### Mortality after 30 days

**FIGURE 1:**
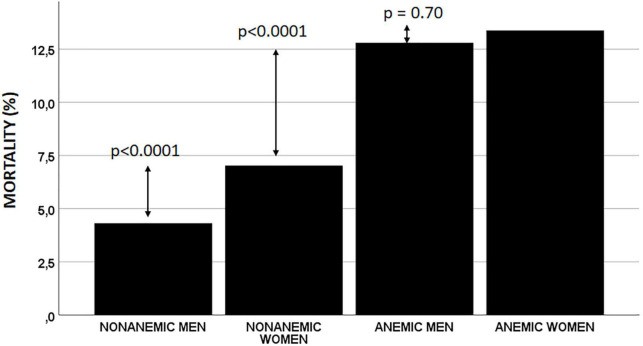
Unadjusted 30-day mortality.

After 30 days, 607 (7.3%) patients had died. The lowest all-cause mortality was observed in non-anemic men (4.3%), followed by non-anemic women (7.0%). Mortality was higher in both anemic men and women (12.8 and 13.4%, respectively) but was similar in both these groups (Table 2 and Figure 1).

After adjustment for confounding factors, the anemia/sex groups were independently associated with 30-day mortality (p = 0.033). Compared with non-anemic men, non-anemic and anemic women had a similar risk of death at 30 days (OR 1.12; 95% CI 0.83–1.52; p = 0.45 and OR 1.30; 95% CI 0.94–1.79; p = 0.11, respectively). However, the anemic men had a 50% higher risk of dying within 30 days than the non-anemic men (OR 1.50; 95% CI 1.13–1.98; p = 0.004) (Table 3).

**TABLE 3:** Predictors of 30-day and long-term mortality (non-anemic men as the reference group).

	30-day mortality		Long-term mortality	
	OR (95% CI)	p	HR (95% CI)	p
Age	1.05 (1.03–1.06)	<0.0001	1.054 (1.050–1.059)	<0.0001
Diabetes	1.36 (1.09–1.72)	0.008	1.52 (1.39–1,66)	<0.0001
Hypertension	0.52 (0.42–0.65)	<0.0001	0.83 (0.77–0.91)	<0.0001
Hyperlipidemia	0.48 (0.36–0.62)	<0.0001	0.74 (0.68–0.81)	<0.0001
Cardiogenic shock	13.05 (9.91–17.17)	<0.0001	2.97 (2.59–3.40)	<0.0001
Anemia/sex group		0.033		<0.0001
Non-anemic women	1.12 (0.83–1.52)	0.45	0.85 (0.76–0.96)	<0.011
Anemic women	1.30 (0.94–1.79)	0.11	1.08 (0.93–1.21)	0.37
Anemic men	1.50 (1.13–1.98)	0.004	1.46 (1.31–1.63)	<0.0001
STEMI	2.97 (2.34–3.77)	<0.0001	1.18 (1.10–1.28)	<0.0001
Renal dysfunction	2.39 (1.91–2.99)	<0.0001	1.84 (1.68–2.02)	<0.0001
P2Y12	0.21 (0.17–0.29)	<0.0001	0.64 (0.57–0.71	<0.0001
TIMI 0/1	2.42 (1.79–3.27)	<0.0001	1.58 (1.38–1.80)	<0.0001
Bleeding	2.17 (1.68–2.80)	<0.0001	1.82 (1.63–2.05)	<0.0001

HR, hazard ratio; OR, odd ratio; P2Y12, P2Y12 receptor antagonists; STEMI, ST-elevation myocardial infarction; TIMI, thrombolysis in myocardial infarction.

Anemic men had a 50% higher risk of dying in 30 days compared to non-anemic men when we compared only males (OR 1.50; 95% CI 1.12–2.00; p = 0.006). However, anemic women had a similar 30-day mortality risk compared to non-anemic women (OR 1.17; 95% CI 0.83–1.64; p = 0.37). When we compared only anemic men and anemic women, they had a similar risk of death at 30 days (OR 0.89; 95% CI 0.65–1.20; p = 0.43).

#### Long-term mortality

**FIGURE 2:**
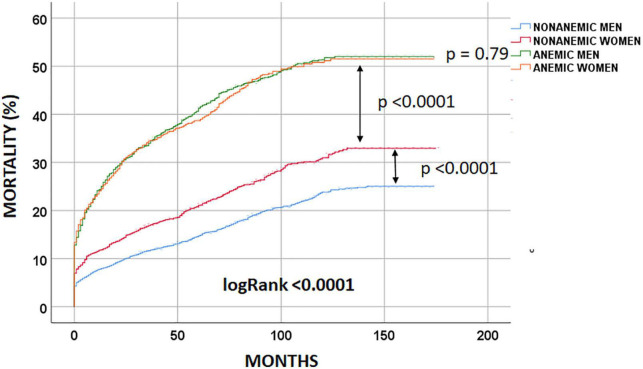
Unadjusted long-term mortality.

At the end of the follow-up period, 2,301 (27.7%) patients had died. Non-anemic men had the lowest long-term observed mortality [803 (18.7%) patients died], followed by non-anemic women [432 (25.3%); logRank < 0.0001 patients died]. Anemic men and women had similar long-term mortality rates [657 (46.2%) and 409 (45.6%); logRank = 0.79 patients had died, respectively] (Figure 2).

**FIGURE 3:**
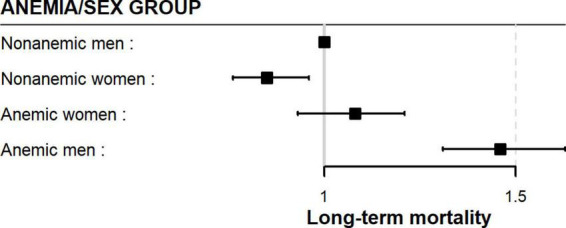
Long-term adjusted mortality risk (non-anemic men as the reference group).

After adjustment for confounding factors, the anemia/sex groups were associated with long-term mortality risk (p < 0.0001) (Table 3). We compared the groups using non-anemic men as a reference. The multivariable-adjusted mortality risk was highest in anemic men. They had a 46% higher risk compared with non-anemic men (HR 1.46; 95% CI 1.31–1.63; p < 0.0001). Non-anemic women had a 15% lower risk of long-term death than non-anemic men (HR 0.85; 95% CI 0.76–0.96; p = 0.011). Moreover, anemic women had a similar long-term mortality risk to non-anemic men (HR 1.06; 95% CI 0.93–1.21; p = 0.37). This suggests that anemia was much less harmful in women than in men in the long term (Table 3 and Figure 3). Considering only men in the analysis, anemic men (compared to non-anemic men) had a 47% higher multivariable-adjusted long-term risk of death (HR 1.47; 95% CI 1.31–1.64; p < 0.0001). Among women, anemic women (compared to non-anemic women) had a 25% higher multivariable-adjusted risk of long-term death (HR 1.25; 95% CI 1.08–1.44; p = 0.002), indicating that anemia is almost twice as dangerous in men as in women. When we compared only anemic men with anemic women, we also found that anemic women had a 24% lower multivariable-adjusted risk of dying in long-term (HR 0.76; 95% CI 0.65–0.87; p < 0.0001), again suggesting that anemia is less harmful in women than in men.

## Discussion

Data on the possible differential impact of anemia on survival in MI patients regarding sex are very rare, conflicting, and inconclusive (6–8, 19). In our long-term follow-up study, we followed patients for up to 14 years, with a median of 7 years. The most important findings of our analysis are as follows:

1. We found a significantly higher unadjusted all-cause mortality rate in anemic men and women compared to their non-anemic peers.
2. The anemic men and women had similar unadjusted mortality rates (Table 2 and Figure 2).
3. Anemic men had twice the multivariable-adjusted long-term mortality risk of women compared with their non-anemic peers (47% higher risk in anemic men and 25% higher risk in anemic women).
4. Anemic women had a similar risk of dying in the long term compared to non-anemic men (Figure 3).
5. Non-anemic women had a 15% lower risk of dying in long-term than non-anemic men (Figure 3).
6. Anemic women had a 24% lower long-term risk of dying in long-term compared to anemic men.

Our results suggest that the influence of anemia in patients with MI is different in men and women. Anemia seems to be much more harmful in male than in female patients with MI in the long term. This was a surprising finding, which, however, is consistent with previous observations in patients with MI (7, 29). In addition, the 30-day mortality risk was only associated with anemia in men.

These results raise several unanswered questions for discussion. Our results contrast with that of Mamas et al. who found a similar association between anemia and outcome in both men and women (17). However, the different nature of the treatment, covariates in multivariable adjustment, and the observation time must be considered when comparing patients. Only 38% of their patients underwent PCI compared to all our patients, which we believe could explain the different results. Our result cautiously strengthens the finding of Tsujita et al. who found a different association between anemia and outcome in men and women (7). In both analyzes, patients were treated invasively. According to our data, the influence of anemia is much stronger in men. In addition, anemic women had a similar risk of long-term death compared to non-anemic men (Figure 3).

The exact mechanisms by which anemia affects mortality after MI remain unclear (7, 30). Anemic patients were more likely to be older and to have diabetes. They also had poorer renal function, less successful PCIs, and higher bleeding rates – all known factors associated with the outcome. We conclude that anemic patients (both men and women) were in poorer health, which may partly explain the higher crude mortality in these patients. Nevertheless, the anemia/sex groups were independently associated with outcome after multivariable adjustment, supporting the hypothesis of a differential effect of anemia in men and women at MI.

Anemia decreases oxygen delivery to the body and myocardium (8, 9, 14, 15, 30). Anemia also results in decreased blood viscosity, which can lead to increased venous return and consequently increased preload (9). Anemic patients compensate by increased systemic vasodilation, decreased systemic vascular resistance, increased stroke volume, and increased heart rate (9, 18, 30–32). Myocardial oxygen demand increases but cannot be met in anemia because the oxygen supply is already decreased, resulting in a mismatch between myocardial oxygen supply and demand (8, 32, 33). In addition, redistribution of the blood flow from the subendocardial to epicardial layers worsens the subendocardial oxygen supply (6). In the presence of coronary stenosis and the setting of myocardial infarction, anemia directly exacerbates myocardial ischemia by reducing oxygen delivery to the injured and jeopardized myocardium (30, 33).

Consistent with our previous report, we found that more women were anemic on admission (34.4% of women and 24.9% of men; p < 0.0001) (8). They also had a “lower reserve” of becoming anemic on admission (124.9 ± 16.8 g/L hemoglobin in women and 139.3 ± 19.4 g/L; p < 0.0001) in association with greater blood loss during hospitalization [3.0 g/L hemoglobin (0.0, 11.0) in women and 1.0 g/L hemoglobin (0.0, 9.0); p < 0.0001]. Consequently, more women were anemic at discharge (47.6% of women compared with 33.6% of men; p < 0.0001).

The most intriguing finding of our analysis was that anemic women had a similar long-term multivariable-adjusted mortality risk to non-anemic men (Figure 3). Compared with non-anemic men, anemic women were older, more likely to have diabetes, hypertension, hyperlipidemia, cardiogenic shock on admission, more extensive and severe coronary artery disease, and a lower success rate of PCI. They also received fewer P2Y12 receptor antagonists but bled more (Table 2). Nevertheless, their long-term mortality risk was similar to that of the non-anemic men. It is unclear why females are more resistant to the deleterious effects of anemia than males. The significantly lower long-term mortality risk of non-anemic women (Figure 3) further supports the previously observed protective effect of the female sex and that the higher observed mortality is the result of additional comorbidities, heart failure, age, risk factors, and bleeding (34, 35).

In addition, anemic women had a 24% lower long-term multivariable-adjusted risk of death than anemic men. They were also older, more likely to have diabetes and hypertension, had more renal dysfunction, and bled more. This finding also suggests that anemia is less harmful in the long term in women than in men.

There are certain female-specific factors, such as possible intrinsic differences in angiogenesis and collateralization between men and women, sex-specific biology due to the protective role of circulating estrogens on vascular endothelium, disease manifestation, unusual pathophysiological mechanisms of coronary artery disease in women, female-specific cardiovascular risk factors (like gestational diabetes), and gender-specific psychosocial stressors (20–23, 36, 37). However, these differences do not explain this result. The various causes of anemia may have different effects on prognosis in patients with MI (occult disease, malignancy, and kidney dysfunction) and these may be the main reason for the worse outcome (7, 8, 30). In addition, we lack data on the long-term course of the anemia during follow-up. Different causes of anemia in men than women and more severe anemia due to underlying disease could explain our result regarding all-cause mortality. Unfortunately, the cause of anemia was not known in our patients.

Another explanation of why women seem to tolerate anemia better could be hormonal changes in the premenopausal period. Due to menstruation, and pregnancies, premenopausal women are more frequently exposed to anemia than men. Menstrual bleeding and anemia during pregnancy are not known to be associated with higher cardiac mortality. Perhaps women’s hearts adapt to anemia during the premenopausal period, and this helps them to tolerate anemia better than men in the postmenopausal period. However, we could not find any data on this issue in the literature.

Ariza-Solé et al. found a different relationship between anemia and outcome in MI patients in different age groups. Anemia was associated with cardiac mortality only in patients aged ≥75 years. However, anemia was significantly associated with all-cause mortality in all patient groups, further blurring the significance of anemia in MI patients (30).

In contrast to most studies, no difference was found between the sexes in reperfusion therapy, so this cannot be the reason for the different association between anemia and outcome in men and women. Consistent with previous observations, we found that bleeding rates were higher in both anemic men and women compared with their non-anemic peers, and anemic women bled even more frequently (Table 2) (7, 8, 15, 25, 27). This would again suggest that women tolerate anemia better in the long term.

There is an ongoing debate as to whether anemia is simply a marker of sicker patients or an independent predictor of adverse clinical outcomes. In our analysis, anemia was clearly observed more frequently in patients with advanced age and comorbidities, suggesting a possible confounding effect in the association between anemia and mortality. However, this increased risk remained after adjustment for potential confounding factors.

Our data have several potential clinical implications. Given the close association between anemia and outcome in both sexes, we hypothesize that an early prophylactic strategy in patients with MI could provide significant clinical benefit. In particular, this includes preventing the worsening of anemia during treatment (radial access to prevent bleeding, careful administration of GP IIb/IIIa receptor antagonists and triple antithrombotic therapy, and avoiding multiple blood draws in the intensive care unit). Future research is needed to determine whether and how to treat anemia in these patients. There are promising data on parenteral iron supplementation in patients with heart failure, but not in patients with MI (38). In addition, a search for occult bleeding may be warranted in these patients, especially in men.

Our results point to the complex relationship between anemia and mortality in MI patients and suggest that it may be difficult to account for all confounding factors that may be present in patients with anemia. The difference in the association between anemia and outcome in men and women may be due to a specific sex-related factor not yet identified or due to unknown confounders not included in our analysis.

Our data confirmed previous finding that anemia is associated with worse outcome in both sexes in long-term. However, our results also suggest that the female sex is more resistant to anemia, making women less susceptible to the deleterious effects of anemia in the long term compared with men. It should be noted that our analysis only shows the association between the sex/anemia group and outcome and does not establish a causal relationship. Possible pathomechanisms are unclear, and in this study, we did not attempt to investigate the mechanism behind this phenomenon, but we did propose several hypotheses. Further research is needed to determine the pathophysiological mechanisms of the different natural course of anemic women and men with MI.

### Limitations

There are limitations to this study. This analysis was a retrospective study at a single center, which allows hypothesis generation but not causality determination. Our data included all-cause mortality only, and the specific contribution of cardiac and non-cardiac causes to all-cause mortality was not examined in detail. Although the association between anemia/sex group and outcome was assessed with a multivariable model, other potential significant confounders may exist that were not accounted for. In addition, the regression adjustments may not ensure balance in the distribution of age or other baseline characteristics. The cause of the anemia was unknown. There are no data on the evolution of anemia during the follow-up period and on how these temporal trends relate to subsequent outcomes. Data on previous coronary artery disease, previous PCIs and surgical revascularization, lung disease, smoking, obesity, hyperuricemia, glucose, and pain-to-balloon time were not available for a sufficient number of patients to be included in the analysis. Data on PCI complications and medication (except for P2Y12 receptor antagonists) after PCI were not available. Data on heart rate and blood pressure, known variables with a strong influence on mortality, were also missing. Finally, there were no exclusion criteria regarding concomitant diseases, so this population represents a real experience of high-risk patients requiring PCI.

## Conclusion

Anemia was detrimental in both sexes. However, our results suggest that anemia is more detrimental in men than in women with MI. Anemia also leads to worse outcomes in women, although to a lesser extent than in men. Whether this result is sex-dependent or due to an as yet unidentified sex-specific factor or unknown confounders that were not accounted for in our analysis remains to be determined.

## Data availability statement

The raw data supporting the conclusions of this article will be made available by the authors, without undue reservation.

## Ethics statement

Ethical, governance, and waiver of consent approvals were granted by the University Medical Center Maribor Committee for Medical Ethics (Reference: UKC-MB-KME-59/19) and all methods were performed in accordance with relevant guidelines and regulations. Written informed consent for participation was not required for this study in accordance with the national legislation and the institutional requirements.

## Author contributions

VK: data collection, study design, analysis, interpretation of data, and writing the first draft of the manuscript. GK: data collection and revision of the manuscript. DS: interpretation of data and revision of the manuscript. All authors discussed the results and contributed to the final manuscript.
